# Real-time detection of Chinese cabbage seedlings in the field based on YOLO11-CGB

**DOI:** 10.3389/fpls.2025.1558378

**Published:** 2025-04-03

**Authors:** Hang Shi, Changxi Liu, Miao Wu, Hui Zhang, Hang Song, Hao Sun, Yufei Li, Jun Hu

**Affiliations:** ^1^ College of Engineering, Heilongjiang Bayi Agricultural University, Daqing, China; ^2^ Heilongjiang Province Conservation Tillage Engineering Technology Research Center, Daqing, China

**Keywords:** Chinese cabbage seedlings, YOLO11-CGB, real-time detection, deep learning, average temperature weight

## Abstract

**Introduction:**

Accurate application of pesticides at the seedling stage is the key to effective control of Chinese cabbage pests and diseases, which necessitates rapid and accurate detection of the seedlings. However, the similarity between the characteristics of Chinese cabbage seedlings and some weeds is a great challenge for accurate detection.

**Methods:**

This study introduces an enhanced detection method for Chinese cabbage seedlings, employing a modified version of YOLO11n, termed YOLO11-CGB. The YOLO11n framework has been augmented by integrating a Convolutional Attention Module (CBAM) into its backbone network. This module focuses on the distinctive features of Chinese cabbage seedlings. Additionally, a simplified Bidirectional Feature Pyramid Network (BiFPN) is incorporated into the neck network to bolster feature fusion efficiency. This synergy between CBAM and BiFPN markedly elevates the model’s accuracy in identifying Chinese cabbage seedlings, particularly for distant subjects in wide-angle imagery. To mitigate the increased computational load from these enhancements, the network's convolution module has been replaced with a more efficient GhostConv. This change, in conjunction with the simplified neck network, effectively reduces the model's size and computational requirements. The model’s outputs are visualized using a heat map, and an Average Temperature Weight (ATW) metric is introduced to quantify the heat map’s effectiveness.

**Results and discussion:**

Comparative analysis reveals that YOLO11-CGB outperforms established object detection models like Faster R-CNN, YOLOv4, YOLOv5, YOLOv8 and the original YOLO11 in detecting Chinese cabbage seedlings across varied heights, angles, and complex settings. The model achieves precision, recall, and mean Average Precision of 94.7%, 93.0%, and 97.0%, respectively, significantly reducing false negatives and false positives. With a file size of 3.2 MB, 4.1 GFLOPs, and a frame rate of 143 FPS, YOLO11-CGB model is designed to meet the operational demands of edge devices, offering a robust solution for precision spraying technology in agriculture.

## 1 Introduction

Chinese cabbage (*Brassica rapa* subsp. *pekinensis*) is the most widely grown and productive vegetable in China, and has been introduced in many countries because of its high yield, ease of cultivation and rich nutrition (Fu et al., 2023). However, pest and disease infestations in Chinese cabbage can significantly diminish its quality and yield, impacting economic returns. Often, these infestations are detected only after extensive damage, missing optimal control opportunities (Wei et al., 2021). To mitigate this, preemptive measures, such as the early application of pesticides during the seedling stage, are common. Nevertheless, conventional spraying methods fail to discriminate between crops and vacant spaces, leading to excessive pesticide application in non-crop areas. This not only results in financial losses but also poses a threat to the food safety of Chinese cabbage (Liu et al., 2023). Excessive pesticide usage also contributes to environmental contamination, soil degradation, and the development of pesticide-resistant pest populations, further complicating agricultural management (Hu et al., 2021). The development of new plant protection equipment based on precision application technology can provide an important guarantee for pesticide reduction and promote the improvement of pesticide utilization, and the key point of this technology lies in how to carry out rapid and accurate detection of Chinese cabbage seedlings. Achieving accurate detection is paramount for optimizing pesticide usage, minimizing environmental harm, and promoting sustainable agricultural practices (Ong et al., 2023; Shi et al., 2023).

The advancement of convolutional neural networks (CNNs) has significantly enhanced deep learning applications in crop identification and detection (Fan et al., 2021). Deep learning-based target detection algorithms are divided into two categories: the two-stage frameworks, which initially generate candidate frames and subsequently classify them using CNNs (Li Z, et al., 2021), and the one-stage frameworks, exemplified by the YOLO series, which employ regression analysis for rapid target detection without needing candidate frames.

The two-stage approach, including algorithms like R-CNN and Faster R-CNN, offers high accuracy but lacks real-time efficiency. For instance, Zhang et al. (Zhang Z, et al., 2023) achieved improved detection accuracy with an enhanced Faster R-CNN for safflower filaments, adaptable to diverse environments. However, the computational burden and model size of this two-stage framework are substantial. Similarly, Vi Nguyen Thanh Le et al (2021). utilized Faster R-CNN for detecting field weeds among various vegetables, achieving satisfactory accuracy but with a high inference time of 0.38 seconds per image, limiting its real-time application potential.

In contrast, the one-stage YOLO series algorithms, as demonstrated by Jin et al. (2022) in vegetable detection, offer speed but sometimes suffer from inaccuracies due to occlusion and proximity issues. Zhang et al. (Zhang Y, et al., 2023) improved YOLOv5 to detect Achnatherum splendens, achieving a high mean Average Precision (mAP) of 95.0% with the largest model, YOLO-Sp-X. However, its size of 740.5MB creates significant computational demands, especially for on-board robotic systems. The smaller model, YOLO-Sp-N, while only 50.4MB, offers a lower mean Average Precision of 81.2%. Wang et al. (2022) integrated the CBAM module into YOLOv5 for real-time detection of Solanum rostratum Dunal seedlings. While effective in test sets, field tests revealed a decline in confidence levels and detection leakages, indicating the need for further refinement. Zheng et al. (2023) designed an intermittent herbicide spraying system for open field kale. Although successful in tests, its model’s limitation to vertical angle shots of kale plants significantly restricts the system’s movement speed, affecting herbicide application effectiveness.

In the field of cabbage detection, Ma et al. (2023) proposed an improved U-Net-based semantic segmentation model, MSECA-UNet, which demonstrated superior detection performance. However, the model requires 64.85 ms for single-image detection, and its image capture perspective is limited to a vertical sample angle, which restricts its potential for rapid robotic movement. Ye et al. (2023) developed a deep learning model, Mask R-CNN, for cabbage crop extraction using unmanned aerial vehicles (UAVs), achieving commendable results. Nevertheless, the model’s lengthy detection time and large size limit its applicability in real-time detection tasks. Sun et al. (2024) introduced the Cabbage Transplantation State Recognition Model Based on Modified YOLOv5-GFD, and Jiang et al. (2024) designed a detection model, YOLOv8-cabbage, for precise cabbage spraying. Both models exhibit a recall rate significantly lower than their accuracy, suggesting potential issues with missed detections. Additionally, these studies also employed a vertical sample angle for image capture.

Current research on cabbage detection, as well as detection of other crops, reveals critical issues that can be observed in similar studies. Overly large and computationally intensive models present challenges for robotic deployment. Additionally, detection models with low precision and recall rates result in frequent omissions and misdetections. The limited and homogeneous datasets hinder model generalization, leading to suboptimal accuracy in practical applications. Furthermore, datasets restricted to vertical sample angles limit the operational speed of machinery. These challenges not only hinder the effectiveness of pest and disease control but also raise broader concerns regarding the economic and environmental impacts of inefficient pesticide use. Inaccurate detection models may lead to excessive pesticide application, contributing to environmental pollution, pesticide resistance, and the loss of biodiversity. To address these challenges, this study proposes an optimized Chinese cabbage seedling detection model based on an enhanced YOLO11 framework. This model integrates several innovations aimed at improving accuracy, speed, and efficiency, making it suitable for real-world agricultural robotic applications. Key improvements include refining the backbone and neck network structures of YOLO11 and implementing a lightweight improvement strategy to reduce computational burden. The model was trained on a comprehensive dataset that includes varying heights, angles, occlusions, and potential environmental interferences, ensuring robust performance in dynamic field environments. By addressing both the technical limitations of current models and the practical challenges in field applications, this study aims to provide a solution that not only enhances detection accuracy but also supports the goals of precision agriculture, reducing pesticide use and minimizing environmental impact.

## 2 Materials and methods

### 2.1 Data acquisition

The dataset used in this study for Chinese cabbage was sourced from Wucuofang Village, Yangcao Town, Anda City, Suihua City, Heilongjiang Province. The collection focused on Chinese cabbage seedlings, which were planted in a single ridge with healthy growth. Image acquisition was carried out using an Honor 30 Pro smartphone, capturing high-resolution images (4096×3072 pixels) in JPEG format. The device featured an f/1.8 aperture and automatic shutter speed adjustment, ensuring proper exposure in various shooting environments.These features contributed to high-quality images with clear details and minimal noise, providing a strong foundation for model training and reliable detection performance.

The f/1.8 aperture used in this study provided a shallow depth of field (DoF) while maintaining sufficient light intake, which is crucial for capturing bright and clear images in low-light conditions. A larger aperture increases the amount of light entering the lens, helping to reduce image noise and blur caused by insufficient lighting, which is particularly important when capturing high-quality images of the Chinese cabbage seedlings under varying natural lighting conditions (such as early mornings, evenings, or overcast days). This aperture setting also helped to separate the Chinese cabbage seedlings from the background, enhancing the visibility of the target object. Furthermore, it emphasized the details of the seedlings, which is beneficial for learning the distinctive features of the target.The automatic shutter speed adjustment feature allowed the device to select the optimal shutter speed based on ambient light, ensuring neither overexposure nor underexposure and minimizing the risk of motion blur. This feature ensured optimal exposure across various lighting conditions, making the features in the captured images more distinct and easier for model detection.

**Figure 1:**
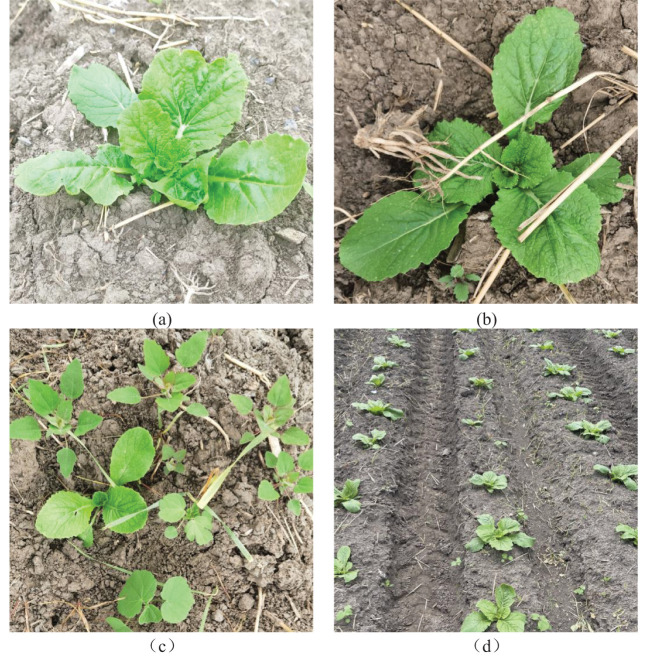
Acquisition of chinese cabbage images. **(a)** A close-up view focusing on a single target. **(b)** An image depicting the subject shaded by straw. **(c)** An image showing interference from a dense weed population. **(d)** A long-distance shot capturing multiple targets.

The data collection period spanned from early August to early September 2023, with image capture times ranging from 06:00 to 18:00. During image acquisition, the distance between the camera and the Chinese cabbage seedlings was controlled between 30cm and 100cm. The shooting angle ranged from 45° to 90° relative to the horizontal direction. The Chinese cabbage seedlings were set as the foreground, while weeds and other elements were considered as background information. A total of 2715 raw images were collected, as shown in **Figure 1**. These images included various data on background complexity, shooting angles, distances, and the number of targets per image, with a particular focus on capturing images of weeds that share similar characteristics with Chinese cabbage seedlings.

### 2.2 Image enhancement

**Figure 2:**
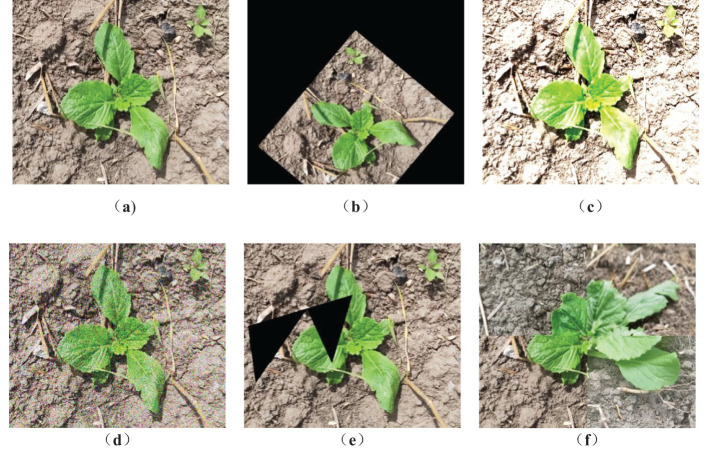
Image enhancement. **(a)** Original image. **(b)** Affine transformations. **(c)**Color distortion. **(d)** Gaussian noise addition. **(e)** Cutout. **(f)** Mosaic.

To ensure the diversity of the Chinese cabbage dataset produced in this study, we employed data augmentation techniques on the collected images. After segregating the dataset into training, testing, and validation sets, the original images underwent several augmentation processes. These included cropping and affine transformations (comprising linear image modifications like translation, rotation, and scaling), color distortion (altering image attributes such as brightness, contrast, saturation, and hue), Gaussian noise addition, Cutout, and Mosaic. These methods, exemplified in **Figure 2**, were instrumental in enriching the dataset’s diversity. Consequently, this expansion significantly enhances the model’s robustness and its ability to generalize.

### 2.3 Dataset construction

In this research, the LabelImg tool, a rectangular region labeling utility, was employed to manually annotate the captured images, pinpointing the exact locations of the target Chinese cabbage seedlings. The annotated data was saved in a.txt file format, culminating in the creation of the Chinese cabbage seedling dataset. The dataset was then randomly divided into training, validation, and test sets in an 8:1:1 ratio, resulting in 2687 training images, 336 validation images, and 335 test images. Each subset contained images of the Chinese cabbage seedlings along with their associated labels. Every image in the dataset includes at least one Chinese cabbage seedling, collectively contributing to a total of 10069 labels.Additionally, the dataset includes detailed information about the shooting height and angle for each image, as detailed in **Table 1**. This comprehensive approach in dataset preparation enhances the precision and efficacy of the deep learning and convolutional neural network analyses that follow.

**Table 1:** Dataset details.

Height\Angle	90°	75°	45°
30cm	385	363	343
70cm	389	381	350
100cm	388	380	379

### 2.4 Chinese cabbage seedling detection model YOLO11-CGB

#### 2.4.1 Network architecture

The YOLO11 algorithm, released by Ultralytics in September 2024, represents a significant evolution in the YOLO series. YOLO11 builds upon the foundation of previous YOLO versions, introducing new features and improvements aimed at enhancing both performance and flexibility. YOLO11 adopts an improved backbone and neck architecture, which strengthens feature extraction capabilities and improves object detection accuracy, especially for complex tasks. Compared to the YOLOv8 model, YOLO11 replaces the C2F module with the C3K2 module, increasing the model’s flexibility and configurability. It continues to utilize the Spatial Pyramid Pooling (SPPF) module, which enhances accuracy while simplifying the model (Tang et al., 2023). Additionally, a C2PSA module is added after the SPPF to further enhance the model’s feature extraction capabilities. YOLO11 retains the Path Aggregation Network-Feature Pyramid Network (PAN-FPN) structure in the neck, which strengthens multi-scale feature fusion (Li et al., 2023). Furthermore, YOLO11 incorporates the head design ideas from YOLOv10, using depthwise separable convolutions to reduce redundant computations and improve efficiency. Compared to previous versions of YOLO models, YOLO11 demonstrates superior performance (Sapkota et al., 2024).

**Figure 3:**
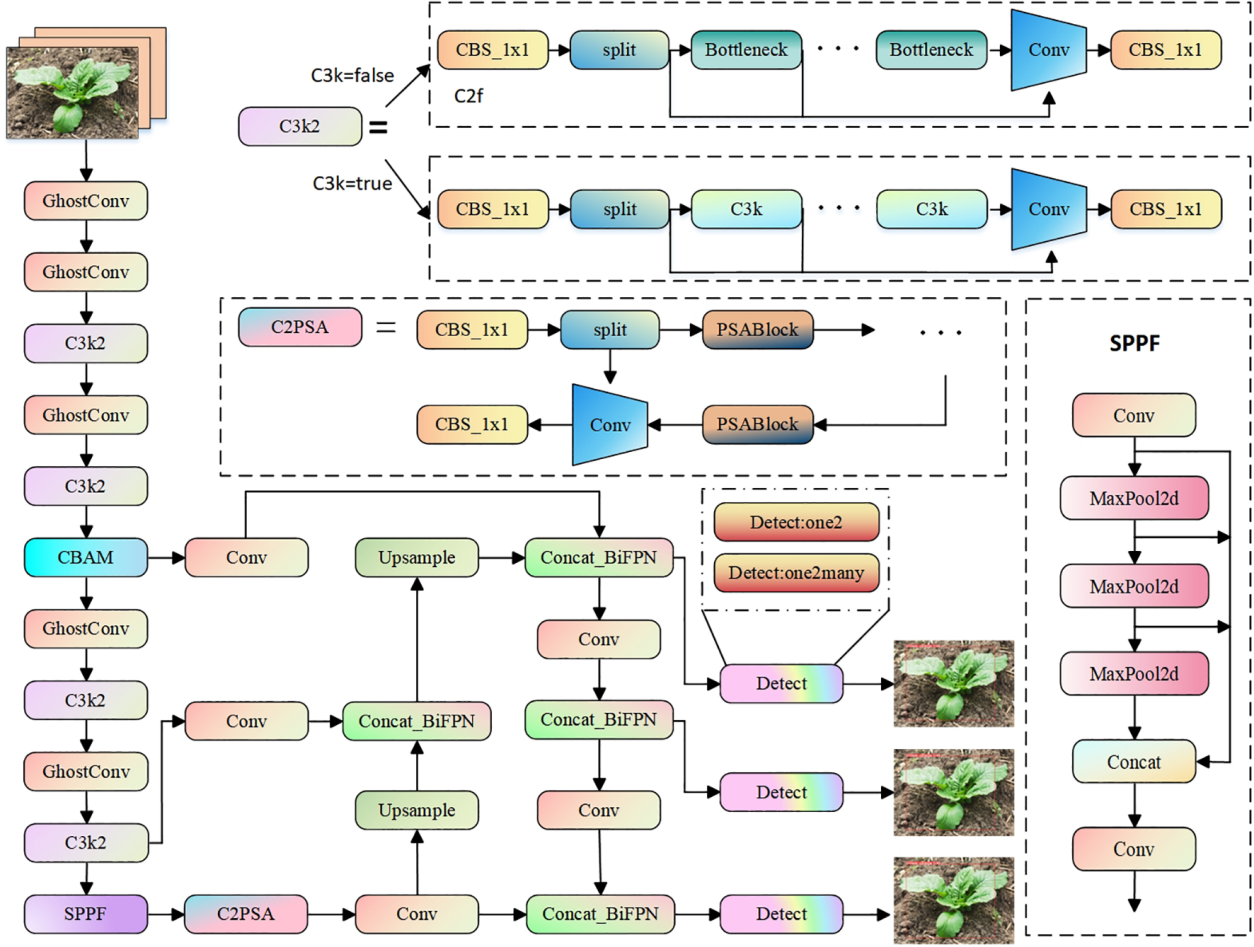
YOLO11-CGB network structure diagram.

In this study, the YOLO11-CGB network model was developed as an enhancement of the YOLO11 nano variant (YOLO11n). This modified version integrates several innovative components to address the challenges of Chinese cabbage seedling detection. The Convolutional Block Attention Module (CBAM) is incorporated to enhance the model’s focus on the distinct features of Chinese cabbage seedlings, ensuring that these key features receive greater attention even in complex backgrounds. The Weighted Bi-directional Feature Pyramid Network (BiFPN) optimizes the network’s feature integration capability by facilitating efficient fusion of features across different scales, which is particularly critical for detecting small and distant seedlings. To reduce the computational burden, GhostConv is employed to significantly shrink the model’s size and computational complexity while maintaining high detection accuracy, making it well-suited for deployment on edge devices. Together, these components enable YOLO11-CGB to not only capture Chinese cabbage seedling features more effectively and extract relevant information with greater precision, but also achieve a balanced reduction in computational and parameter complexity, enhancing its practical applicability. The structure of the improved network is illustrated in **Figure 3**.

#### 2.4.2 CBAM attention mechanism

**Figure 4:**
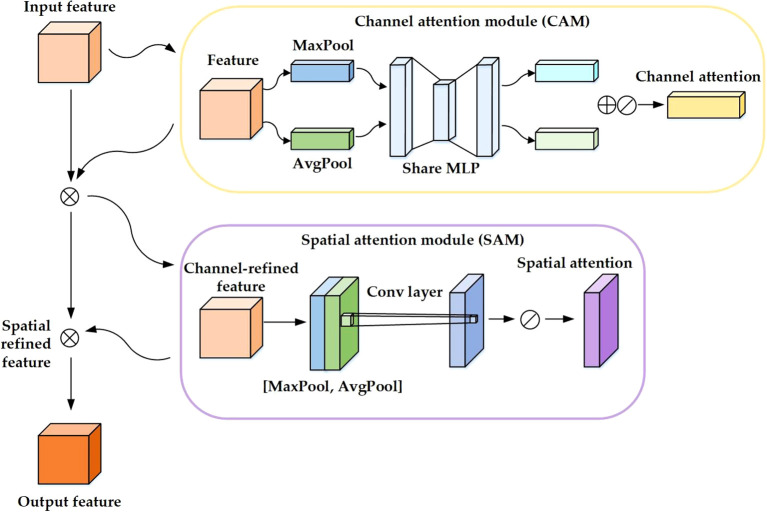
CBAM network structure diagram.

The Convolutional Block Attention Module (CBAM) is a streamlined and efficient attention mechanism for feed-forward convolutional neural networks (Woo et al., 2018; Karim et al., 2024). Its lightweight structure enables seamless integration into convolutional neural network frameworks, facilitating end-to-end training with the base convolutional neural network. CBAM comprises two primary components: the Spatial Attention Module (SAM) (Fu et al., 2017) and the Channel Attention Module (CAM) (Hu et al., 2020), as depicted in **Figure 4**.

In the context of identifying features of Chinese cabbage seedlings, the SAM plays a pivotal role. It performs a transformation process in the spatial domain of the image, extracting crucial feature information that is vital for accurate detection. Concurrently, the CAM is responsible for assigning appropriate weight coefficients to the feature channels based on their relative importance. This dual approach ensures a more focused and relevant feature extraction, contributing significantly to the model’s overall detection efficacy.

One of the primary challenges in field detection of Chinese cabbage seedlings is distinguishing them from weeds that share similar characteristics. To overcome this challenge, YOLO11-CGB integrates the CBAM attention mechanism after the C3K2 module in the third layer. This approach allows the network to retain more low-level details, enhancing its feature extraction capabilities for targets with similar characteristics., effectively improving the model’s precision.

#### 2.4.3 Ghostconv

**Figure 5:**
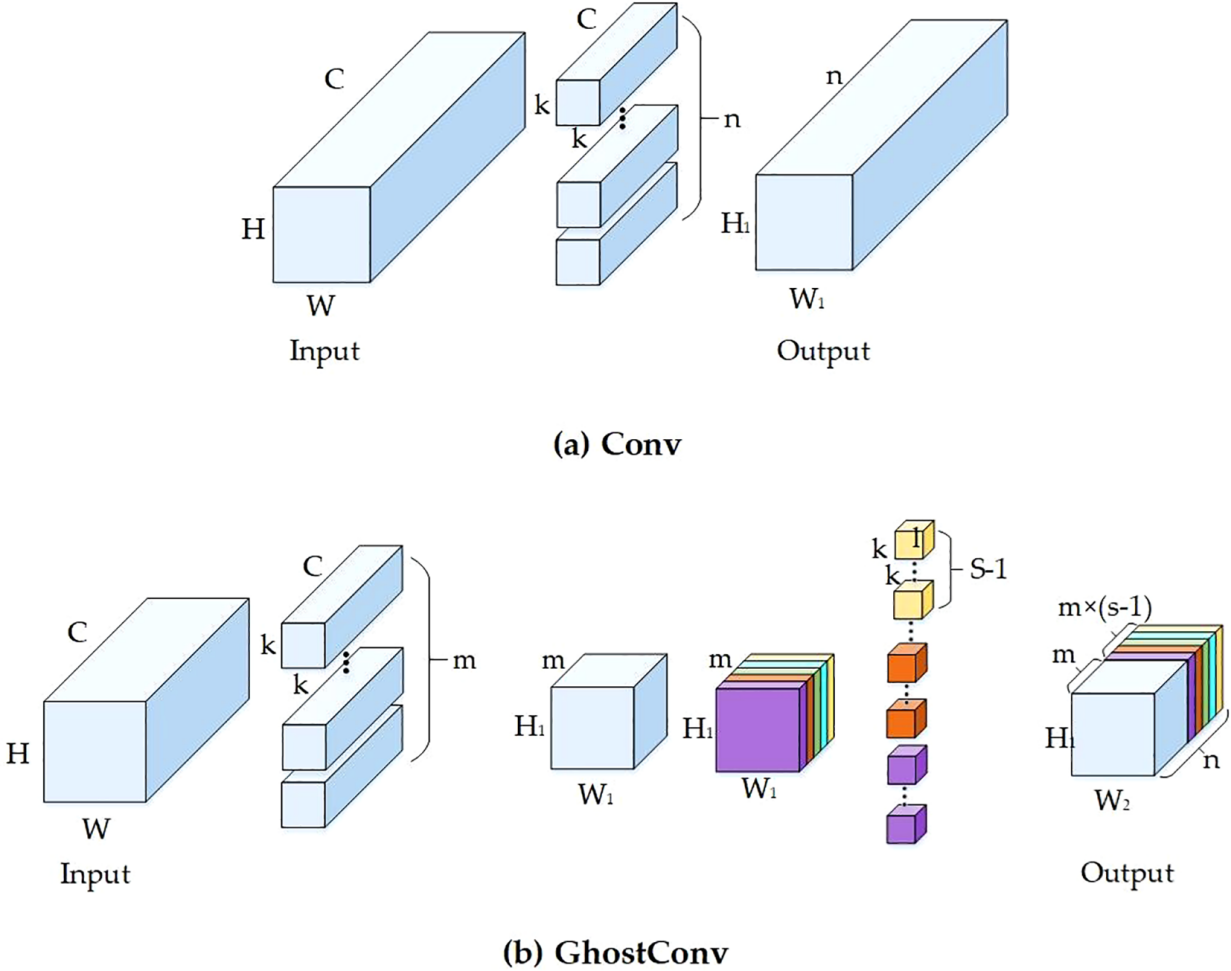
Structure of convolution module. **(a)** Conventional convolution module. **(b)** Ghostconv convolution module.


In the YOLO11 network, the convolutional layer tends to consume substantial memory during feature extraction, as illustrated in **Figure 5a**. However, with the increasing application of convolutional neural networks (CNNs) in embedded devices, there is a heightened demand for reduced memory usage and enhanced computational efficiency in neural networks. GhostConv, an innovation originating from Huawei’s Noah’s Ark Laboratory’s GhostNet, is a lightweight network that effectively minimizes computational resource demands while preserving accuracy. It leverages the redundancy in feature maps to conduct cost-efficient linear transformations, as illustrated in **Figure 5b** (Han et al., 2020; Wei et al., 2020). GhostConv operates distinctly from traditional CNNs, functioning in two primary stages. Initially, it employs standard convolutional processes to produce a feature map. This map, though channel-sparse, is information-rich. Subsequently, the feature map count is augmented through computationally efficient methods, which, when merged with the initial maps, form the final output. Essentially, GhostConv bifurcates the conventional convolution process. It begins with operations using a limited number of convolution kernels, followed by channel-level convolutions using smaller kernels (e.g., 3×3 or 5×5). These are then concatenated with the output from the first stage.The parameters required for GhostConv include the height (h), width (w), and the number of channels (c) for the input features; and for the output features, the height (H), width (W), number of convolution kernels (n), kernel size (k), size of the linear transformation kernel (d), and the number of transformations (s). Additionally, rs and rc represent the computational and parametric ratios of standard convolution to GhostConv convolution, as delineated in Equations 1 and 2.

(1) $$\begin{array}{c} r_{s}=\frac{h\times w\times c\times H\times W\times n}{\frac{n}{s}\times H\times W\times k\times k\times c+(s-1)\times \frac{n}{s}\times H\times W\times d\times d}\\ =\frac{c\times k\times k}{\frac{1}{s}\times c\times k\times k+\frac{\left(s-1\right)}{s}\times d\times d}\approx s \end{array}$$

(2) $$r_{c}=\frac{n\times c\times k\times k}{\frac{n}{s}\times c\times k\times k+(s-1)\times \frac{n}{s}\times d\times d}\approx \frac{s\times c}{s+c-1}\approx s$$

Taking into account both Equations 1 and 2, it becomes evident that the ratio of computation to the number of parameters is intricately linked to the number of transformations, denoted as ‘s’. This implies that the model’s speedup is more pronounced as the quantity of generated feature maps escalates. Consequently, the incorporation of GhostConv convolution within the model markedly diminishes both the computational and parameter demands. This reduction directly translates to an increase in the model’s execution speed and overall computational efficiency, making it an invaluable adaptation for optimizing convolutional neural network operations.

YOLO11-CGB replaces the convolutional modules in the backbone network with GhostConv modules, effectively reducing the model’s weight. The neck network, however, still utilizes traditional convolutional modules, which enhances the model’s multi-scale feature fusion capabilities. This improvement strategy ensures that the model maintains its detection performance while increasing efficiency and practicality. It also reduces the number of parameters and computational complexity, making the model more suitable for deployment on resource-constrained platforms, such as edge devices and embedded systems.

#### 2.4.4 BiFPN structure

**Figure 6:**
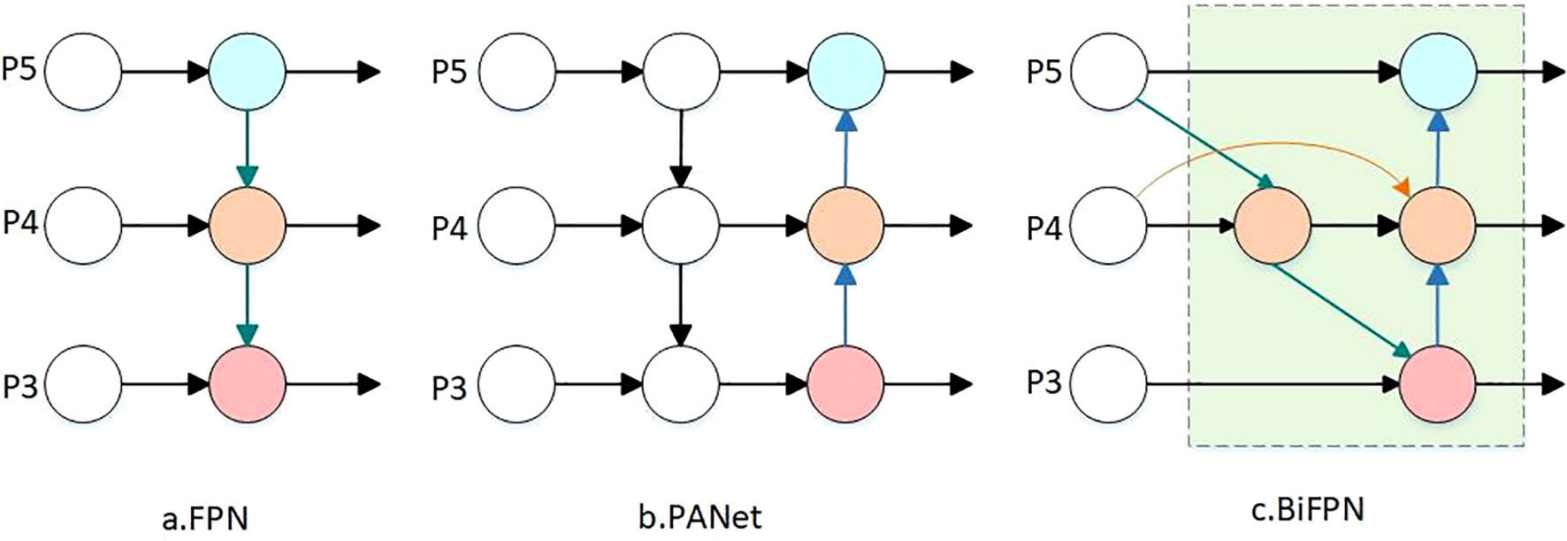
Diagram of the three network architectures. **(a)** Traditional FPN network architecture. **(b)** PANet network structure. **(c)** BiFPN network architecture.

In tasks involving multi-scale feature fusion, the conventional Feature Pyramid Network (FPN) typically employs a top-down approach for fusing various input features. However, this methodology often results in significant loss of shallow feature information during the transfer process (Xiao et al., 2022). In contrast, the Path Aggregation Network (PAN) utilized by the YOLO11 model, while based on FPN’s design, introduces additional bottom-up pathways. This bidirectional fusion approach within the backbone network facilitates more effective propagation of lower-layer information, although its structure remains relatively simple (Dong et al., 2022). In our study, we incorporate the Bidirectional Feature Pyramid Network (BiFPN) (Tan et al., 2020), which allows for weighting, to address these limitations. The architecture of the three Neck networks, including this enhanced BiFPN, is illustrated in **Figure 6**. This integration of BiFPN in the model structure significantly augments the effectiveness of feature fusion, ensuring a more balanced integration of both deep and shallow features.

BiFPN deletes the single input node with small contribution to simplify the network, then adds an edge between the original input node and the output node to fuse more features, and finally fuses the top-down and bottom-up paths into a module, which is designed as a parameter into the network after calculating the number of repetitions of the module by NAS technology to improve the accuracy of the feature extraction of the Chinese cabbage seedling in order to realize the feature fusion at a higher level.

The improvement to the neck network not only enhances the model’s ability to fuse multi-scale features but also improves the efficiency and effectiveness of the feature fusion process. The updated network architecture allows the model to maintain efficient computation while better capturing features from targets of different scales. This is particularly advantageous for small target detection in complex scenarios, where the model demonstrates significant improvements.

### 2.5 Test platform and parameters

The experimental framework for this study was executed on the following platform specifications: model training tasks were performed using the aotudl cloud computing server, equipped with a Intel(R) Xeon(R) Platinum 8352V CPU @ 2.10GHz, an RTX 4090 (24GB) GPU. The operating system used was Ubuntu 18.04 (64-bit), and the deep learning framework utilized was PyTorch 3.8.0 with CUDA 12.4.

In this study, the network optimization was conducted using the SGD optimizer. The settings included a batch size of 32 and a total of 300 epochs for iterations. The initial learning rate was set at 0.007, and the cosine annealing strategy was employed as the learning rate decay optimization technique. This particular strategy enables a gradual reduction of the learning rate by modulating it in the form of a cosine function, effectively preventing the model from converging to a local optimum. The underlying mathematical principle of the cosine annealing strategy is delineated in Equation 3. This approach ensures a more refined and effective optimization process, crucial for the robust performance of the network.


(3) $$\eta_{t}=\eta_{\min}+\frac{1}{2}(\eta_{\max}-\eta_{\min})\left(1+\cos\left(\frac{T_{cur}}{T_{\max}}\pi \right)\right)$$

where T_cur_ denotes the current round of training, T_max_ denotes the total number of rounds of training, η_max_ and η_min_ denote the maximum and minimum values of the learning rate, respectively.

The primary advantage of this strategy lies in its dynamic adjustment of the learning rate: initially, it decreases rapidly, facilitating swift convergence of the model in the early training phase. Subsequently, in the latter stages of training, the rate of decrease in the learning rate slows down. This gradual reduction allows for more meticulous parameter adjustments, thereby enhancing the model’s ability to generalize. This careful balance between rapid initial convergence and slower, more precise fine-tuning in later stages is crucial for optimizing the model’s overall performance and effectiveness.

### 2.6 Evaluation indicators

To thoroughly evaluate the performance of the model presented in this paper, a range of metrics has been employed. These include precision (P), recall (R), F1 score (F1), mean Average Precision at IoU 50 (mAP50), Giga Floating-Point Operations Per Second (GFLOPs), the total number of parameters (Parameters), and Frames Per Second (FPS).

In this case, precision and recall are used as the basic metrics, and mAP is used as the final evaluation metric to measure the recognition correctness of the model.

Precision is the proportion of correctly predicted positive samples out of all samples predicted as positive. It measures the accuracy of the model’s predictions for the positive class. Specifically, it evaluates how many of the instances identified as Chinese cabbage seedlings by the model are indeed correctly identified, ensuring that the predictions for the target class are accurate. The definition is shown in Equation 4:


(4) $$\begin{array}{c} Precision=\\ \frac{\text{Number of Correctly Identified Chinese Cabbage Seedlings}}{\text{Number of Correctly Identified Chinese Cabbage Seedlings+Number of Incorrectly Identified Chinese Cabbage Seedlings}} \end{array}$$

Recall is calculated based on the proportion of all targets correctly predicted and examines the ability of the model to find all positive samples. The definition is shown in Equation 5:


(5) $$\begin{array}{c} Recall=\\ \frac{\text{Number of Correctly Identified Chinese Cabbage Seedlings}}{\text{Number of Correctly Identified Chinese Cabbage Seedlings}+\text{Number of Missed Chinese Cabbage Seedlings}} \end{array}$$

The F1 score is the harmonic mean of Precision and Recall, providing a balanced metric that considers both false positives and false negatives. it combines the precision and recall into a single number to give a more comprehensive evaluation of the model’s performance. The definition is shown in Equation 6:


(6) $$F1=\frac{2\times Precision\times Recall}{Precision+Recall}$$

Mean Average Precision (mAP) is a commonly used metric in object detection that combines precision and recall by averaging the precision across multiple recall values. mAP50 refers to the mAP calculated with an Intersection over Union (IoU) threshold of 0.50. This metric is useful for evaluating the overall detection accuracy, considering both the correct identification of objects and the precision of their localization.

GFLOPs serve as an indicator of the complexity of the model or algorithm. In contrast, parameters reflect the model’s size. Generally, lower values of parameters and GFLOPs signify reduced computational demands, facilitating easier deployment to end devices and less stringent hardware requirements. FPS denotes the number of frames processed by the model per second, a critical metric in real-time applications. The FPS value is influenced not only by the algorithm’s weight but also by the hardware configuration of the experimental device.

## 3 Results and analysis

### 3.1 Training results

**Figure 7:**
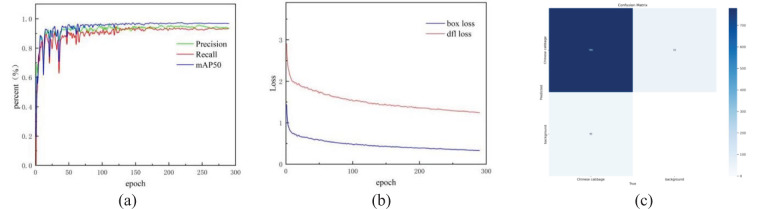
YOLO11-CGB network training. **(a)** changes in precision, recall and mAP50. **(b)** changes in Box Loss and Dfl Loss. **(c)** Confusion matrix diagram of YOLO11-CGB.

In this study, the Early Stopping training strategy was employed, meaning that training was terminated early if no improvement was observed over the past 50 epochs. The YOLO11-CGB model underwent a total of 290 iterations. **Figure 7a** illustrates the changes in precision, recall, and mAP50 throughout the training process. During the initial 75 iterations, precision, recall, and mAP50 exhibited oscillatory growth before gradually stabilizing. After 125 iterations, the model parameters no longer showed significant oscillations, and after 200 epochs, mAP50 stabilized, indicating that the model was approaching saturation and key performance metrics became stable. **Figure 7b** shows the loss curves for bounding box regression (Box Loss) and Distribution Focal Loss (Dfl Loss). It can be observed that the loss function rapidly converged in the early stages and gradually flattened. Both Box Loss and Dfl Loss steadily decreased, and no significant fluctuations were observed after 200 epochs, suggesting that the training process was stable without overfitting. The final YOLO11-CGB model achieved a precision of 0.947, a recall of 0.93, an F1 score of 0.938, and an mAP50 of 0.97. The model contains 3.2M parameters, with a GFLOPS value of 4.1, and a processing speed of 143 samples per second.

**Figure 7c** displays the confusion matrix for the model, which correctly identified targets in 780 samples, demonstrating the model’s strong detection capability. This result provides strong evidence of the effectiveness of the proposed method in object detection tasks. The number of false positives was only 55, indicating that the model was able to accurately distinguish between targets and the background in most cases, with a low false positive rate. The number of false negatives was 40, demonstrating that the model successfully detected the majority of target samples, resulting in an extremely low miss rate. Furthermore, the model showed high robustness in handling complex target-background scenarios. This outcome validates the reliability of the proposed method in real-world applications.

### 3.2 Comparative performance tests of different models

To validate the detection capability of the improved YOLO11-CGB model, its performance was compared with five widely-used object detection models: Faster R-CNN, YOLOv4, YOLOv5s, YOLOv8n, and YOLO11n, using the Chinese cabbage seedling dataset. To further demonstrate the model’s performance, several recent representative studies on crop seedling detection and Chinese cabbage detection were also compared. These included the Seedling-YOLO for broccoli seedlings (Zhang T, et al., 2024), the improved YOLOv8 for maize seedlings (referred to as YOLOv8-Maize) (Liu et al., 2024), the improved YOLOv7 for Chinese cabbage seedlings (referred to as YOLOv7-CCSB) (Gao et al., 2024), the YOLOv8-cabbage specifically designed for Chinese cabbage (Jiang et al., 2024), and the YOLOv8-Ghost-Backbone for mature Chinese cabbage (Zhang H, et al., 2024). The model training results are summarized in **Table 2**.

**Table 2:** Comparative performance test results.

Model	P	R	F1 score	mAP50	GFLOPs	FPS	Parameters
Faster R-CNN	86.60%	87.70%	87.10%	89.20%	196.7	21	137M
YOLOV4	89.90%	88.90%	89.40%	91.60%	83.9	37	55M
YOLOV5s	91.60%	87.20%	89.40%	92.90%	18.2	43	14M
YOLOV8n	91.30%	89.40%	90.30%	93.40%	8.2	83	6.0M
YOLO11n	93.70%	91.20%	92.40%	94.60%	6.3	111	5.5M
Yolo11-CGB	94.70%	93.00%	93.80%	97.00%	4.1	143	3.2M
Seedling-YOLO	91.30%	92.10%	91.70%	94.30%	11.6	30	5.0M
YOLOv8-Maize	92.90%	87.00%	90.00%	93.40%	-	45	-
YOLOv7-CCSB	91.30%	83.40%	84.30%	84.30%	-	60	-
YOLOv8-cabbage	95.50%	85.10%	90.00%	93.90%	-	38	-
YOLOv8-Ghost-Backbone	93.40%	92.50%	92.90%	94.40%	9.2	83	6.5M

As shown in **Table 2**, compared with five commonly used models in the field of object detection, namely Faster R-CNN, YOLOv4, YOLOv5s, YOLOv8n, and YOLO11n, the YOLO11-CGB model demonstrates superior performance in the detection of Chinese cabbage seedlings in terms of precision, recall, and mean Average Precision (mAP). Specifically, the precision is improved by 1.0% to 8.1%, recall is increased by 1.8% to 5.8%, and the F1 score rises by 1.4 to 6.7. These improvements highlight the progress of YOLO11-CGB in reducing both missed detection and false detection rates. Furthermore, mAP50 increases by 2.4% to 7.8%, reflecting the enhancement of model performance, robustness, and exceptional generalization ability. These improvements in precision, recall, and mAP can be attributed to the integration of the CBAM attention mechanism, which effectively enhances feature extraction and representation, as well as BiFPN’s efficient fusion of multi-scale feature information. Additionally, a comparative analysis of GFLOPs, FPS, and model parameters shows that YOLO11-CGB has significantly lower computational load than the other models. This efficiency is largely due to the lightweight nature of the GhostConv module and the simplified neck network structure, which substantially reduces computational demands and model size, facilitating easier deployment of the improved model on mobile devices or embedded systems.

Moreover, compared to other models targeting different crop seedlings and specifically those targeting Chinese cabbage, YOLO11-CGB also demonstrates relatively outstanding performance. Compared to the five cited models, YOLO11-CGB leads in F1 score and mAP50 to varying degrees, indicating its superior detection performance among similar models. Additionally, YOLO11-CGB has significantly lower GFLOPs and model parameters, with a considerable advantage in FPS compared to other models, reflecting its efforts in lightweight design. This also demonstrates the model’s efficiency in computation and storage, verifying its suitability for real-time detection tasks on field-edge devices.

### 3.3 Ablation test

To ascertain the validity of the enhancements incorporated in the YOLO11-CGB model, we conducted a series of ablation tests. These tests were designed to evaluate the individual and collective impacts of each improvement made to the original YOLO11n model. Specifically, we integrated the CBAM attention module into the backbone layer, applied the weighted Bidirectional Feature Pyramid Network (BiFPN) in the neck layer, and replaced the standard convolutional (Conv) module with GhostConv in the network. We conducted tests on the original YOLO11n with each of these improvements independently, as well as in combinations of two or three, to assess the synergistic effects of these modifications.These experiments were carried out under identical conditions using the Chinese cabbage seedling dataset developed for this study. The outcomes, which provide insights into the efficacy and interaction of each enhancement within the YOLO11-CGB model, are systematically presented in **Table 3**.

**Table 3:** Comparison of ablation test results.

Model combination	mAP50	GFLOPs	FPS	Parameters
YOLO11n	94.60%	6.3	111	5.5M
YOLO11n+CBAM	96.40%	6.5	105	5.6M
YOLO11n+BiFPN	95.80%	4.8	137	3.7M
YOLO11n+GhostConv	94.50%	5.4	128	4.5M
YOLO11n+CBAM+BiFPN	97.10%	5	133	3.9M
YOLO11n+CBAM+GhostConv	96.00%	5.5	126	4.7M
YOLO11n+BiFPN+GhostConv	95.80%	3.9	147	3.1M
YOLO11-CGB	97.00%	4.1	143	3.2M

**Table 3** clearly demonstrates that incorporating the CBAM attention module into the backbone network and utilizing the weighted bidirectional feature pyramid network (BiFPN) in the neck layer significantly improves the model’s average accuracy. These enhancements enable the YOLO11-CGB model to achieve higher precision and exceptional detection performance. However, the inclusion of the CBAM attention mechanism increases the number of parameters and computation of the model, which reduces the speed of detection. In contrast, the improved BiFPN network structure, combined with the use of GhostConv to replace the original convolution (Conv) modules, substantially reduces the model’s computational burden and model size, leading to a significant increase in FPS. Remarkably, the model does not suffer from a loss in detection speed due to the lightweight design; on the contrary, it significantly accelerates detection speed, reflecting a substantial enhancement in feature extraction capability under limited computational resources. There is no apparent interference between the three enhancement strategies, and each contributes uniquely when working together. The above experimental data analysis validates the effectiveness of YOLO11-CGB on the large Chinese cabbage seedling dataset.

### 3.4 Performance comparison of models incorporating different attention mechanisms

To explore the impact of different attention modules on the performance of the YOLO11n model, four distinct attention mechanisms were integrated into the YOLO11 network for comparative study. These mechanisms include Squeeze-and-Excitation Attention Module (SE), Channel Attention Module (CA), Simple Attention Module (SimAM), and Convolutional Block Attention Module (CBAM). The resulting models were named YOLO11-SE, YOLO11-CA, YOLO11-SA, and YOLO11-CBAM, respectively. Each attention module was strategically placed before the convolutional layer in the fourth network layer. This positioning was chosen to enhance the model’s feature extraction capabilities, particularly for medium- to small-scale samples. All four models were trained and tested under the same experimental conditions using the same Chinese cabbage seedling dataset, and the results are presented in **Table 4**.

**Table 4:** Performance comparison of models incorporating different attention mechanisms.

Model	Precision	Recall	mAP50
YOLO11n	93.70%	91.20%	94.60%
YOLO11-SE	93.90%	92.00%	95.80%
YOLO11-CA	94.10%	91.60%	95.50%
YOLO11-SA	93.70%	90.90%	95.10%
YOLO11-CBAM	94.20%	92.30%	96.40%

**Table 4** shows that, compared to the original YOLO11n model, the YOLO11 model enhanced with attention mechanisms demonstrates significant improvements in precision, recall, and mAP50, indicating that adding attention mechanisms to the model positively impacts its performance. Among these models, the YOLO11-CBAM model shows the most significant gains. Compared to the original YOLO11n, its precision improves by 0.5 percentage points, recall increases by 1.1 percentage points, and mAP50 rises by 1.8 percentage points. Additionally, the performance of YOLO11-CBAM also surpasses that of the other three attention mechanisms, showcasing that the CBAM attention mechanism provides a more substantial performance enhancement for the model.

### 3.5 Comparison of model improvement results

**Figure 8:**
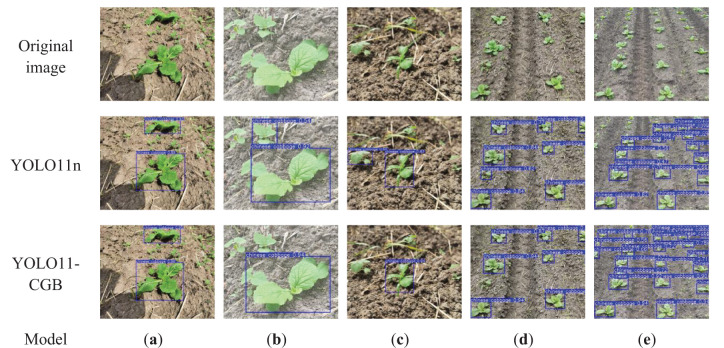
Comparison of model detection results. **(a)** Normal environment. **(b)** Weed interference with similar features. **(c)** Occlusion by weeds. **(d)** Small-angle multi-target scenario. **(e)** Large-angle multi-target scenario.

The original YOLO11n network model and the improved YOLO11-CGB model were tested and compared on the image test set. The test set contains 335 images and 930 Chinese cabbage seedling samples. Representative images from the trained test set were randomly selected for presentation, as shown in **Figure 8**. The false positive and false negative counts in the results were manually counted and compared, and the comparison results are presented in **Table 5**.

**Table 5:** Comparison of model improvement results.

Model	Number of targets detected	Number of missed detections	Probability of missed detection	Number of false detections	Probability of false detection
YOLO11n	833	106	11.4%	9	1.0%
YOLO11-CGB	926	7	0.8%	3	0.3%

As shown in **Figure 8a**, both the YOLO11n and YOLO11-CGB models exhibit commendable performance on the near-distance dataset. Both models demonstrate a certain level of resistance to weed interference, accurately distinguishing Chinese cabbage seedlings from other plant types. However, compared to the original YOLO11n model, the YOLO11-CGB model shows a slightly higher confidence level in detection. In **Figure 8b**, where weeds with features similar to Chinese cabbage are present, the YOLO11n model produces false positives. In contrast, the YOLO11-CGB model, with enhanced feature extraction capabilities and stronger interference resistance, reduces misdetections. As seen in **Figure 8c**, both models are able to recognize Chinese cabbage seedlings occluded by weeds, but the YOLO11-CGB model outperforms YOLO11n in terms of detection confidence, with YOLO11n again misidentifying weeds with features similar to Chinese cabbage. **Figure 8d** shows the detection results under a small-angle scenario, where, after the distance increases, the YOLO11n model produces false positives, while the YOLO11-CGB model accurately distinguishes Chinese cabbage seedlings from weeds due to its strong feature extraction capabilities. **Figure 8e** presents the detection results under a large-angle multi-target scenario. The YOLO11n model’s detection ability significantly decreases, missing some distant Chinese cabbage seedlings. In contrast, the YOLO11-CGB model maintains a high detection rate for distant targets and demonstrates noticeably better detection confidence compared to YOLO11n. These observations collectively highlight the superior performance of the YOLO11-CGB model in various challenging environments.

An analysis of **Table 5** and **Figure 8e** shows that, compared to the original YOLO11n model, the performance of the YOLO11-CGB model has substantially improved, particularly in detecting targets at the far end of the images. The original YOLO11n model had notable issues with missed detections for distant targets. However, the improved YOLO11-CGB model exhibits a significant reduction in the missed detection rate, which dropped by 10.6%, with the final missed detection rate standing at just 0.8%. This enhancement effectively addresses the practical agronomic requirements for real-world applications. Additionally, the false positive rate was markedly reduced from 1.0% to 0.3%, highlighting the optimization of the model’s detection accuracy for distant targets at large angles.

**Figure 9:**
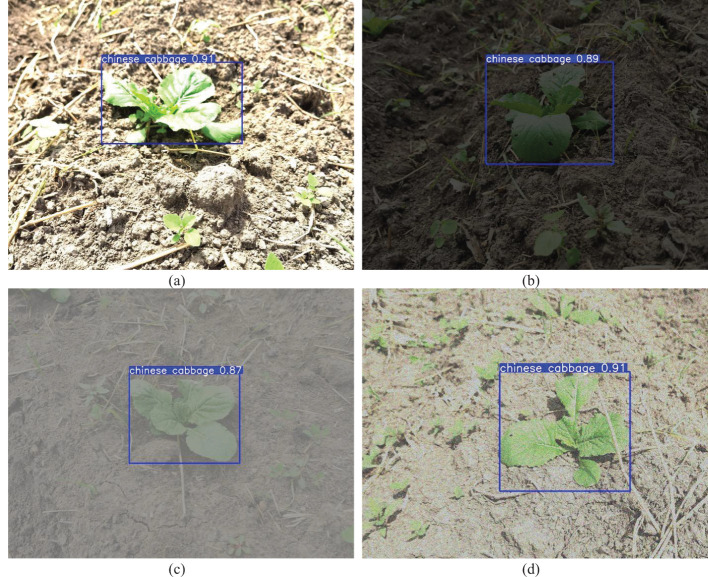
Detection tests under different simulated environmental conditions. **(a)** Direct sunlight scenario. **(b)** Dim lighting scenario. **(c)** Foggy weather. **(d)** Noise interference.

To further validate the adaptability and robustness of the YOLO11-CGB model under varying environmental conditions, image processing techniques were used to simulate various actual agricultural operations scenarios. These included adjusting brightness and saturation to mimic direct midday sunlight and dim evening conditions, as shown in **Figures 9a, b**, respectively. Furthermore, low-contrast extreme environments, such as foggy weather, were simulated by reducing the contrast, as shown in **Figure 9c**. Noise interference, which may occur due to field signal transmission and other factors, was simulated by injecting Gaussian noise, as depicted in **Figure 9d**. The experimental results demonstrate that the model maintains excellent stability and robustness when handling variations in lighting conditions, making it suitable for all-weather detection tasks and ensuring reliable results throughout different times of the day. Even under extreme weather conditions, while the model’s confidence slightly decreased, it still maintained a high detection accuracy overall, showcasing the model’s exceptional environmental adaptability and its ability to handle complex scenarios. This provides a solid technical foundation for achieving precise Chinese cabbage seedling detection in dynamic and changing environments.

### 3.6 Comparison of model visualization effects

**Figure 10:**
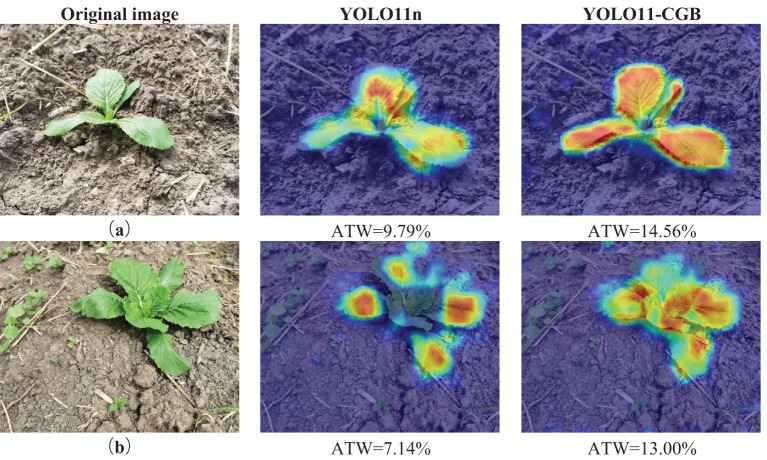
Model detection thermograms. **(a)** Standard state. **(b)** With weed interference.

In this study, we used the class-activation heat map Grad-CAM (Selvaraju et al., 2017; Xie et al., 2022) to visualize and compare the features extracted after convolution of the fourth layer of the YOLO11n and YOLO11-CGB models in Chinese cabbage seedling detection. This visualization reveals the image regions that the convolutional neural network focuses on in the Chinese cabbage seedling detection task, highlights the discriminative part of the image that influences the model’s decision, and makes it more intuitive to see the effect of the network’s feature extraction on different regions of the Chinese cabbage seedling. In the heat map, the color depth of the red region (high temperature region) indicates the importance of this region in the detection process. The comparison results of the class activation heat map are shown in **Figure 10**.

In order to quantitatively evaluate the ability of different models in feature extraction, this study proposes an average temperature weight value (ATW) as a digital reference index to measure the effectiveness of the heat map. When calculating the average temperature weight value: firstly, in order to reduce the interference in the background map, the thermogram is segmented, leaving only the foreground map with thermal range for the calculation of ATW.The color space conversion of the processed image is carried out to convert the image from RGB color space to Lab color space, which is beneficial to weaken the interference of external factors and highlight the color differences. Subsequently, the reference color is defined, and four representative colors in the heat map, namely, red, yellow, green and blue, are selected as the weight benchmarks, and the colors on the heat map are grouped into the four benchmark colors according to the principle of approximation.Each base color is assigned a weight for the temperature it represents in the heat map, with red representing the highest temperature with a weight of 1.0, and red and its neighboring colors proportionally assigned a weight of 0.8 to 1.0. Yellow and its similar color family are proportionally assigned a weight of 0.6 to 0.8; green and its similar color family are assigned a weight of 0.4 to 0.6; and blue, as the lowest temperature representative, is assigned a weight of 0. On this basis, the CIEDE2000 color difference formula is used to compute the color difference between each pixel of the image and the reference color in Lab space, and the dynamic weight interpolation function is used to compute the weight values.The ATW value is obtained by accumulating the whole image pixel weights and dividing by the total number of whole image pixels. The higher ATW finally obtained indicates that the overall temperature of the image is higher; on the contrary, it indicates a lower temperature, which effectively reflects the differences in the temperature distribution of the thermogram and provides a quantitative means for the comparison of the model efficacy.The formula of ATW is shown in Equation 7.


(7) $$ATW=\frac{1}{N}\sum_{i=1}^{N}\left(\sum_{c\in C}w_{ic}\cdot I(d_{ic},c)\right)$$

where N is the total number of pixels in the image, C is a set of predefined reference colors, dic denotes the CIEDE2000 color difference between the ith pixel and the reference color c, wic denotes the weight of each color C on each pixel i calculated based on the color difference dic, and I(dic,c) is an exponential function with the value of 1 when the color c is the closest reference color of the pixel i, and the otherwise the value is 0.

Analysis of **Figure 10a** reveals that the YOLO11-CGB model exhibits a significantly larger activation area on Chinese cabbage seedling features compared to the YOLO11n model, with the color tending towards higher temperatures. This indicates that YOLO11-CGB responds more sensitively and effectively to features than its YOLO11n counterpart. From **Figure 10b**, it can be observed that the activation area of YOLO11n is more focused on the leaves, representing the model’s tendency to capture leaf features. In contrast, YOLO11-CGB focuses on the overall features of the Chinese cabbage seedling, with a more comprehensive activation area, thereby enhancing the model’s generalization. This improvement reduces the impact of environmental variables and provides better resistance to weed interference in both models.

The Average Temperature Weight (ATW) values of YOLO11-CGB are notably higher than those of YOLO11n in both figures. Specifically, there is a 4.77% increase in ATW in **Figure 10a**, corresponding to a 32.8% enhancement, and a 5.86% increase in ATW in **Figure 10b**, equating to a 45.1% enhancement. These results signify that the network modifications have markedly bolstered the extraction of key features within the images. The improved ATW values reflect a substantial enhancement in the network’s capacity to discern and process critical information, with a heightened focus on pivotal regions. Additionally, these results demonstrate the network’s improved sensitivity, enabling it to more effectively differentiate between crucial features and background noise, thereby augmenting the overall performance of the model.

## 4 Discussion

YOLO11-CGB can realize the task of fast and accurate inspection of Chinese cabbage seedlings, and the volume of the model and the detection speed are both limited to a superior range. Most of the precision spraying robots in the current research capture images in vertical direction (Ye et al., 2023; Sun et al., 2024; Jiang et al., 2024), this image capture method can reduce the pressure of the detection algorithm, but it will likewise limit the movement speed of the robot, resulting in many precision spraying robots being limited to 0.5m/s (Zheng et al., 2023; Hu et al., 2022).

This study not only incorporates images in the vertical direction, but also focuses on high and large-angle distal Chinese cabbage seedling recognition, and the better distal recognition effect can enable the model to buy more time for the robot after being applied to the precision spraying robot, providing favorable support for improving the robot’s operation speed.

In future research, we will continue to further develop the Chinese cabbage seedling detection model, focusing on expanding the number and variety of weeds in the dataset to enhance the model’s resistance to a broader range of weeds. Additionally, we will incorporate a target tracking function into the model to prevent the repeated spraying of the same Chinese cabbage seedling during pesticide application. We will deploy the model on mobile devices, integrating precision spot-spraying equipment with an autonomous walking chassis, thus providing further technical support for the research of precision spraying robots.

## 5 Conclusions

1. To enhance the detection of Chinese cabbage seedlings in agricultural settings, we propose an advanced YOLO11-CGB model. This model incorporates a Convolutional Block Attention Module (CBAM) within its backbone network, augmenting its capability to discern key features amidst complex backgrounds. Additionally, the neck network employs a simplified Bidirectional Feature Pyramid Network (BiFPN), which effectively boosting feature fusion efficiency, thereby enhancing detection accuracy, reducing the model’s computational load, and increasing detection speed.A notable innovation in the YOLO11-CGB model is the substitution of standard convolutional modules in the backbone network with GhostConv modules. This adjustment markedly diminishes the model’s size and optimizes computational efficiency without compromising accuracy. The optimized model has a compact parameters of just 3.2 MB and a computational demand of 4.1 GFLOPS, rendering it highly suitable for real-world deployment in precision spraying robots.
2. To rigorously assess the YOLO11-CGB model’s efficacy, we curated a specialized dataset of Chinese cabbage seedlings captured in natural settings. This dataset encompasses a diverse array of images showcasing seedlings from various angles and heights, set against backgrounds with multiple disturbances. We enhanced the dataset using image processing techniques such as affine transformations, color warping, Gaussian noise addition, Cutout, and Mosaic. The YOLO11-CGB model demonstrates exceptional performance on this dataset, achieving a precision of 94.7%, a recall of 93.0%, and a mean Average Precision (mAP) of 97%. These metrics reflect improvements of 1.0%, 1.8%, and 2.4%, respectively, over the baseline YOLO11n model. Additionally, the model boasts a rapid detection speed of 7 milliseconds, underscoring its high accuracy and efficiency in detection tasks.
3. To objectively assess and visualize the class-activated thermograms of the YOLO11-CGB model, both pre- and post-improvement, we introduce an Average Temperature Weight (ATW) as a quantitative metric. This index serves to evaluate the efficacy of the thermograms. Through this approach, we conducted a thorough numerical and visual analysis of the model’s class-activated thermograms. The findings indicate a significant enhancement in the ATW of the improved model, registering an increase of 32.8%-45.1%. This improvement suggests that the YOLO11-CGB model exhibits a more refined feature extraction capability. The outstanding performance of the YOLO11-CGB model not only provides critical technical support for the development of precision spraying robots but also offers valuable insights for the advancement of precision agriculture technologies.

## Data Availability

The raw data supporting the conclusions of this article will be made available by the authors, without undue reservation.
